# Efficacy and Safety of Socheongryong-Tang Among Atopic Dermatitis Patients With Respiratory Disorders: A Double-Blinded, Randomized, Placebo-Controlled Clinical Trial

**DOI:** 10.3389/fphar.2020.597885

**Published:** 2020-11-26

**Authors:** Ju Hyun Lee, Eun Heui Jo, Jee Youn Jung, Young-Eun Kim, Mi-Ju Son, Su Jin Kang, Geum Jin Yang, Yu Hwa Shim, Min Cheol Park

**Affiliations:** ^1^ Department of Korean Medicine Ophthalmology and Otolaryngology and Dermatology, Wonkwang University Korean Medicine Hospital, Iksan, South Korea; ^2^ Department of Acupuncture and Moxibustion, Wonkwang University Korean Medicine Hospital and Research Center of Traditional Korean Medicine, Wonkwang University, Deokjingu, South Korea; ^3^ Clinical Medicine Division, Korea Institute of Oriental Medicine, Daejeon, South Korea; ^4^ Future Medicine Division, Korea Institute of Oriental Medicine, Daejeon, South Korea; ^5^ Department of Korean Medicine Obstetrics and Gynecology, Wonkwang University Korean Medicine Hospital, Iksan, South Korea; ^6^ Korean Medicine Dermatology Clinical Research Center of Wonkwang University, Iksan, South Korea; ^7^ Department of Korean Medicine Ophthalmology and Otolaryngology and Dermatology, Wonkwang University Korean Medicine Hospital and Research Center of Traditional Korean Medicine, Wonkwang University, Iksan, South Korea

**Keywords:** allergy, atopic dermatitis, clinical trials, height growth, socheongryong-tang

## Abstract

Atopic dermatitis is a chronic inflammatory skin disease that affects the growth and development of children. The prevalence of atopic dermatitis has been continually increasing, and this has also been accompanied by rising socioeconomic costs. Interest has been growing in alternative medicine as a means of alleviating the burden of atopic dermatitis. This was a single-center, double-blinded, randomized, placebo-controlled investigator-led clinical trial including 60 atopic dermatitis patients. The participants were classified into an experimental group (30 persons) and a control group (30 persons), who were administered, respectively, socheongryong-tang or a placebo for 4 weeks. After 4 weeks of treatment, the participants visited the trial center again and assess their efficacy and safety. The researchers performed statistical comparisons of the changes in the SCORAD Index, amount and frequency of ointment use, and height and weight to assess the efficacy. To assess the safety, diagnostic tests and vital sign checks were performed at each visit, and the presence or absence of adverse events was observed. As a result, the frequency and the amount of steroid ointment application in both groups increased, but the experimental group showed less tendency (*p* = 0.081). Results of analyzing the children in the experimental group in relation to growth showed a significantly greater height growth than the control group (*p* < 0.05). In addition, all study participants did not show any remarkable abnormal signs in the safety evaluation. In conclusion, compared to the control group, the experimental group, who took socheongryong-tang showed a tendency to be less dependent on steroid ointment and statistically significant increase in height.

## Introduction

Atopic dermatitis (AD, also called atopic eczema) is a chronic relapsing inflammatory skin disorder that is accompanied by itching, is caused by genetic and environmental factors (Nutten, 2015; Otsuka et al., 2017), and is common in infants (Eichenfield et al., 2013). Due to the “allergic march”, patients who suffered from AD in infancy are more likely to develop respiratory disorders such as allergic asthma or rhinitis in childhood (Bantz et al., 2014). Several studies on allergic disease have reported that atopic disease affects the growth and development of children. Although the mechanisms by which allergic diseases impair height growth have not yet been elucidated (Kristmundsdottir and David, 1987), children with a history of respiratory allergy or AD are more likely to be shorter than their peers (Ferguson et al., 1982; Kristmundsdottir and David, 1987; Sant’Anna et al., 1996).

The prevalence of AD has been persistently increasing to date (Deckers et al., 2012; Nutten, 2015), and this has been accompanied by rising socioeconomic costs. There has been growing interest in alternative medicine as a means of reducing the socioeconomic burden of AD. However, excluding a study analyzing the growth-promoting ingredients in boyangsungjang-tang (Hong et al., 2012) or studies demonstrating the efficacy of gamisasangja-tang (Park et al., 2015) and taeumjowi-tang (Park et al., 2019) for alleviating AD, there have been hardly any clinical studies demonstrating the efficacy of alternative medicine for AD and growth.

Socheongryong-tang (SCRT, also known as Sho-Seiru-To and Xiao-Qing-Long-Tang) is a type of herbal medicine that has been used for centuries to treat respiratory and allergic diseases in countries across East Asia (Ko et al., 2004; Das et al., 2009). However, in spite of these effects of SCRT, there have been almost no studies demonstrating its effects on AD and childhood growth. This research aims to evaluate the efficacy and safety of SCRT, vs. placebo, for the treatment of AD with respiratory disorders and as a growth promoter for short children.

## Materials and Methods

### Sample Size Calculation

To evaluate SCRT efficacy in AD patients, we used SCORAD scores. In SCRT and placebo groups, It was hypothesized that the mean SCORAD scores change after intervention will be 7.9 (μc) and 13.8 (μt), respectively, with a standard deviation of 8.2. Based on the estimated dropout rate (20%), 60 participants in total were needed to achieve significance level of 0.05 in a 2-tailed test and a statistical power of 80%.

### Study Design and Participants

This study was designed as a single-center, double-blinded, randomized, placebo-controlled, investigator-led clinical trial. The participants were divided into an experimental group (30 persons) and a control group (30 persons), who were administered SCRT or placebo, respectively, for 4 weeks. After 4 weeks of medication, the participants visited the trial center again, and the efficacy and safety of the treatments were assessed. The trial design of this study has already been published in the form of a protocol paper (Kang et al., 2020). This clinical trial was conducted from June 13, 2019 (first registration date) to December 13, 2019 (last visit date).

### Patient Enrollment

A total of 65 persons were enrolled in the study, of which five persons were excluded during screening. The reasons for exclusion were contravening the exclusion criteria (two persons), contravening the inclusion criteria (two persons), and withdrawal of consent (one person). Investigator-led clinical trials were for trend search rather than statistical and clinical significance, and n number was selected as 30 cases per group considering 20% dropout rate.

### Inclusion and Exclusion Criteria

Among patients who had heard and fully understood a thorough explanation of the study, voluntarily decided to participate, and gave written consent to adhere to the study precautions, those who satisfied all the following inclusion criteria and did not fit any of the exclusion criteria were defined as participants.

#### Inclusion Criteria

1. Male or female individuals aged 7–65 years, having mild-moderate atopic dermatitis (SCORAD score ≥15 and <50), and with ≥3 major clinical symptoms and ≥3 minor clinical symptoms according to the Hanifin & Rajka diagnostic criteria (Hanifin and Rajka, 1980)
2. Individuals who checked ≥1 respiratory disease or related symptom corresponding to the allergy march in the questionnaire for symptom classification

#### Exclusion Criteria

1. Patients receiving intensive drug treatment for severe AD
2. Patients who have used oral antihistamines, steroids, antibiotics, systemic photochemotherapy, or other immune-suppressants less than 4 weeks before the start of the trial
3. Patients with a systemic infection or receiving systemic antibiotic therapy, or taking interferon therapy
4. Patients with severe skin disease or pigment deposition other than AD, or with extensive scarring in the region of AD
5. Patients with liver disease, kidney disease, severe acute cardiovascular disease, gastrointestinal disorders, excessive sweating, voiding dysfunction, or chronic disease
6. Patients with a platelet count ≤100,000/mm^3^ due to liver dysfunction caused by chronic hepatitis
7. Patients who have taken potassium-containing agents, licorice-containing agents, glycyrrhizic acid, salt-containing agents, loop diuretics (furosemide, ethacrynic acid), thiazide diuretics (trichlormethiazide), ephedra/ephedrine-containing agents, MAO inhibitors, thyroid-related drugs, catecholamines, or xanthines less than 4 weeks before the start of the trial
8. Patients who have taken antipsychotic drugs less than 2 months before screening
9. Patients who have participated in another clinical trial within the last 4 months
10. Patients with a history of drug/alcohol abuse, hypersensitivity to any drugs used in this clinical trial or ingredients in those drugs
11. Women who are pregnant, breastfeeding or capable of childbearing (i.e., women who have not undergone surgical sterilization) and do not accept the use of appropriate contraceptive methods
12. Patients with thyroid dysfunction, indicated by thyroid-stimulating hormone (TSH) ≤0.1 or ≥10.0 µU/ml in screening

### Investigated Drug and Placebo

The experimental group took SCRT granules (Batch number: 18423; Hanpoong Pharm and Foods, Jeonju, Jeollabuk-do, South Korea) and the control group took a placebo (Batch number: 18001; Hanpoong Pharm and Foods), which are all water extracted brown granules (Table 1). The participants took SCRT or the placebo three times per day for 4 weeks, taking one sachet before each meal. The one-time dose of SCRT or placebo was determined with reference to the regulations on reporting product licenses for herbal medicines published by the Korean Food and Drug Administration, differentiating according to age. The participants aged 7–14 years took a dose of 6.0 g per day, and the participants aged 15–65 years took a dose of 9.0 g per day.

**TABLE 1:** Ingredients of the SCRT granules and placebo.

	Ingredient name	Amount (g)	Extract (g)		Ingredient name	Amount (g)
Main Ingredient	*Pinellia ternata* (Thunb.) Makino	2.00	1.05	Excipient	Lactose hydrate	1.490
	*Ephedra sinica* Stapf	1.00			Corn starch	1.490
	*Paeonia lactiflora* Pall.	1.00		Colorant	Caramel pigment	0.010
	*Glycyrrhiza uralensis* Fisch.	1.00		Fragrance	Ginseng flavor powder	0.010
	*Cinnamomum cassia* (L.) J. Presl	1.00		—	—	—
	*Asiasarum heterotropoides* var. *mandshuricum* (Maxim.) F. Maek.	1.00		—	—	—
	*Zingiber officinale* Roscoe	1.00		—	—	—
	*Schisandra chinensis* (Turcz.) Baill.	1.00		—	—	—
—				—	—	—
Excipient	Lactose hydrate	—	0.82	—	—	—
	Cornstarch	—	1.13	—	—	—
Total	—	—	3.00	Total	—	3.00

To provide the minimal treatment for AD, all participants were supplied with Lacticare (Hydrocortisone acetate 1%) ointment, which is a Class 7 steroid, corresponding to the classification of “the least potent agent” (Ference and Last, 2009). Lacticare is a white lotion manufactured by Korea Pharma Co., Ltd. (Hwaseong, Gyeonggi-do, South Korea). All the participants were instructed to rub a thin layer of the ointment broadly into the affected area, in accordance with their physician’s guidance. Each drug was stored at room temperature (20–30°C) in a sealed container, and storage, management, and distribution of the drugs was conducted under the guidance of the principal investigator and a traditional Korean medicine physician acting as a trial administrator.

### Randomization and Blinding

#### Randomization

The participants who gave written consent to participate in the trial were allocated a screening number by researchers according to the order in which they gave consent. Of these, participants who satisfied the inclusion/exclusion criteria were assigned a randomization number in the order in which they visited. Treatment prescribed to the participants was based on these numbers assigned to the participants.

The randomization table contains information about the administration group assigned to the participants according to the randomization number. A block randomization method was used to randomize based on a 1:1 allocation ratio within a block of a certain size (e.g., 4, 6, 8). After generating the randomization table, for each participant, the corresponding drug was packaged, a label with the randomization number was attached, and the drug was sent to the pharmacist or traditional Korean medicine physician administrator at the trial center before the start of the trial.

#### Blinding

To ensure scientific results for the trial, double-blinding was performed, so that the investigators and participants were both unaware of the group allocation of each participant. In the event of unblinding due to adverse events, this was to be reported to the IRB and the petitioner was to be informed in accordance with the unblinding protocol.

### Efficacy Assessment

#### SCORAD Index

The SCORAD Index is a scale for assessing AD that was developed in 1996 and has been used to date (European Task Force on Atopic Dermatitis, 1993). The investigators used the SCORAD Index to ascertain objective symptoms, such as the extent and severity of lesions, used the VAS to quantify and calculate subjective symptoms, such as pruritus and sleep disorders. Based on this data, the changes in the SCORAD Index were compared statistically between the two groups.

#### Ointment Application

Drugs that were prescribed for treatment purposes and could affect the results, such as antihistamines, steroids, and NSAIDs, were thoroughly banned. However, in order to provide the minimal treatment for AD, all participants were provided with the steroid ointment Lacticare. The study participants were instructed to apply an appropriate amount of steroid ointment to the skin lesion with a cotton swab whenever atopic dermatitis symptoms occurred. To prevent adverse reactions, the maximum number of applications per day of steroid ointment was limited to 1 to 3 times. The participants recorded their own frequency of ointment use every day in a notebook, and based on this record, they visited the research institute again on Week 4 to calculate the total number of times they used the ointment. The total amount of ointment used was measured using scales when the patients visited the trial center again 4 weeks after the start of the trial. Based on these measurements, the change in ointment use was compared statistically between the two groups.

#### Body Measurement

The investigators measured and recorded the participants’ height and body weight when they visited the trial center at baseline and in Week 4. Based on these measurements, changes in height and weight were compared between the two groups.

### Safety Assessment

#### Adverse Reaction

In this study, all harmful, unintended signs, symptoms, and diseases that the participants developed after receiving a drug in the trial were defined as adverse reactions. In the event of an adverse reaction, the investigators compiled a case record form including the name of the adverse reaction, the date of onset and recovery, by severity, relatedness to the drug taken, related procedures and treatments, and treatment outcomes. Abnormal laboratory results or vital signs that were considered to be clinically significant were also recorded as adverse reactions.

#### Diagnostic Test

For safety assessment, all participants were subjected to laboratory tests for a total of 20 items, including complete blood cell (CBC), renal function test (RFT), liver function test (LFT), blood lipid, and urine PH. The tests were performed twice, at baseline and in Week 4.

#### Vital Sign Monitoring

The investigators measured all participants’ body temperature, pulse rate, and blood pressure when they visited the trial center at baseline and in Week 4. Changes in vital signs were monitored before and after taking the prescribed drug, and judged to be normal or abnormal based on the results.

### Statistical Analyses

SAS for Windows was used for statistical analysis; the statistical significance level was set at 5% and the power at 80%, and 95% confidence intervals were provided for the differences between the groups. For test results, means, standard deviations, and frequencies were shown. Paired *t*-tests were used to compare the efficacy outcome variables between baseline and Week 4, and independent *t*-tests were used to analyze differences between the two groups. However, when normality was not satisfied through the normality test, nonparametric analysis was performed using Wilcoxon signed-rank test and Mann-Whitney test. The participants’ gender and ratio of participants who developed adverse reactions was calculated and compared between the two groups using a chi-square test. The chi-square test, Fisher’s exact test, and independent *t*-test were used for comparative analysis of factors that influence height growth. Means and standard deviations were shown for laboratory test results and vital signs, and independent t-tests and paired t-tests were used to compare the results, respectively, within groups between baseline and Week 4, and between groups. Missing values (e.g., due to dropout) were imputed using last observation carried forward (LOCF).

## Results

### Demographic Information

There were no statistically significant differences in sex or age between the two groups. Statistical analysis in this study included a total of 49 participants, excluding the four participants who dropped out and seven participants who, in a MAST test for around 60 allergens to test for respiratory allergies, diverged from the normal distribution for an item on the statistically significant boundary between the two groups. The seven participants showing differences in the MAST test were all in the experimental group, and showed allergic reactions to garlic (one person), Cladosporium (three persons), *Alternaria alternata* (one person), and dog (two persons).

**FIGURE 1:**
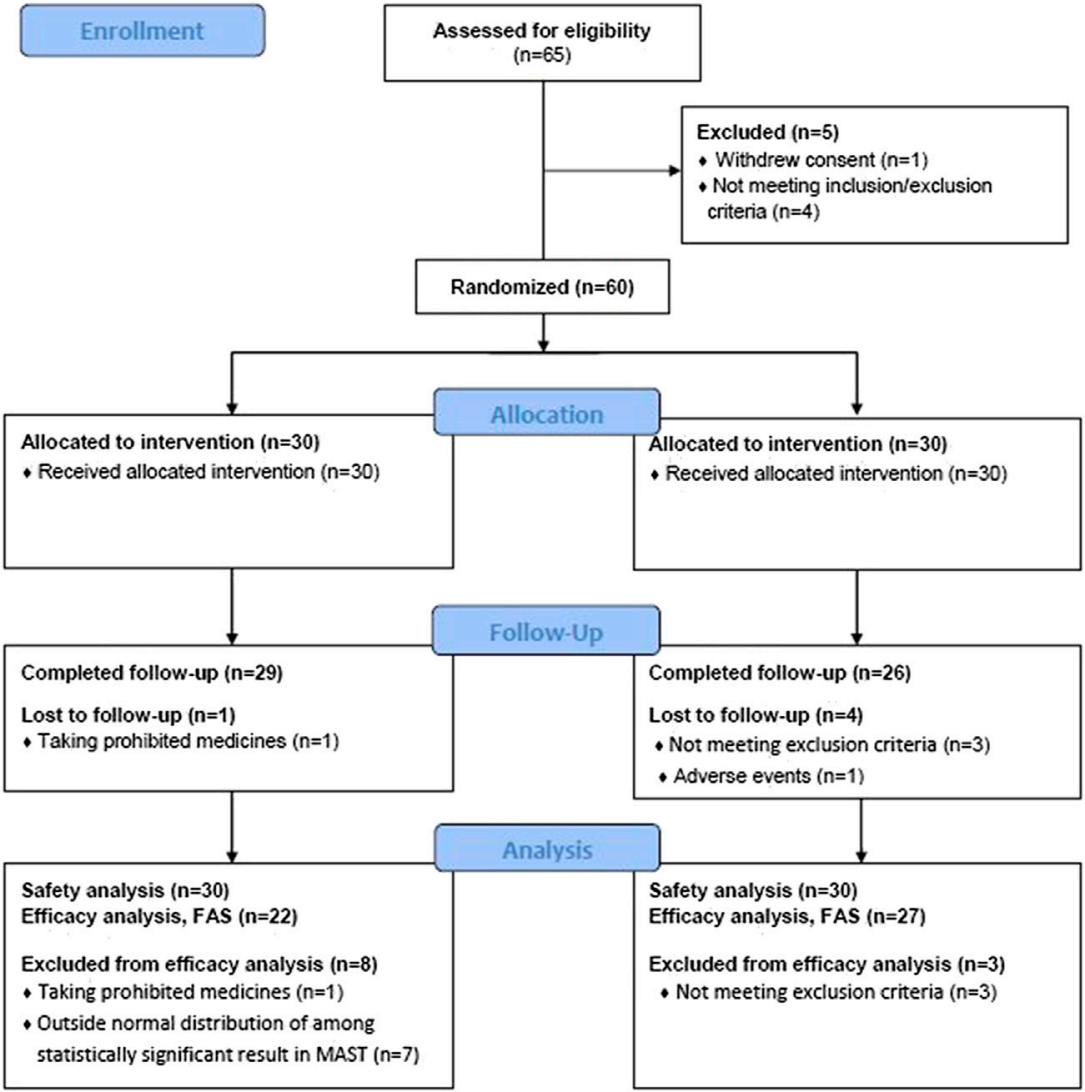
Study flow chart describing the randomized controlled trial.

In order to precisely ascertain the extent of growth, participants who were aged <18 years were classified as children. A total of 30 children were recruited (15 in the experimental group and 15 in the control group). Among the children, there were no statistically significant differences in sex or age between the two groups (Table 2). One person in the experimental group and three persons in the control group dropped out during the trial. The reason for dropout in the experimental group was concomitant medication with a banned drug, and TSH outside the normal range in the placebo group. Among the children, one participant of the experimental group dropped out due to violation of exclusion criteria. Excluding the patients who dropped out, 56 patients completed the whole clinical trial in accordance with the prepared protocol (Figure 1).

**TABLE 2:** Demographic information.

		Experimental group (n = 30)	Control Group (n = 30)	Total (n = 60)	*p*-value^a^
Total participants	Sex (M/F)	16/14	9/21	25/35	0.067^b^
	Age (years)	22.53 ± 14.67	25.47 ± 16.89	24.00 ± 15.75	0.476
		**Experimental group (n = 15)**	**Control group (n = 15)**	**Total (n = 30)**	***p*-value^a^**
Children participants	Sex (M/F)	9/6	6/9	15/15	0.273^b^
	Age (years)	10.00 ± 1.96	10.13 ± 2.10	10.07 ± 2.00	0.859

a Values are presented as mean ± SD: analyzed by independent *t*-test.

b Values are presented as mean ± SD: analyzed by chi-square test.

### Compliance

The experimental group took a mean of 76.97 ± 12.01 doses of the prescribed drug, showing 94.55 ± 5.18% compliance. The control group took a mean of 76.33 ± 18.16 doses of the prescribed drug, showing 93.67 ± 6.21% compliance. Compliance was over 90% in both groups, and there was no statistically significant different between the two groups.

### Efficacy Evaluation

#### SCORAD Index

In the efficacy evaluation in Week 4, the objective, subjective, and total SCORAD scores all showed statistically significant decreases compared to baseline in both the experimental group and the control group (*p* < 0.05). However, there were no significant differences when SCORAD scores were compared between the two groups (Table 3).

**TABLE 3:** Changes in the SCORAD index.

	Experimental group (n = 22)				Control group (n = 27)				*p*-value^b^
	Baseline	Week 4	Change value	*p*-value^a^	Baseline	Week 4	Change value	*p*-value^a^	
Extent of lesion	33.14 ± 12.13	23.77 ± 11.41	−9.36 ± 10.30	0.0003***	32.41 ± 15.86	23.37 ± 12.75	−9.04 ± 8.39	<0.0001***	0.903
Severity of lesion	4.27 ± 1.39	3.18 ± 1.40	−1.09 ± 0.92	<0.0001***	4.19 ± 1.44	3.11 ± 1.45	−1.07 ± 0.83	<0.0001***	0.947
Subjective symptoms	9.36 ± 3.37	6.59 ± 3.32	−2.77 ± 2.49	<0.0001***	8.52 ± 3.62	6.26 ± 3.72	−2.26 ± 2.46	<0.0001***	0.473
Total score	30.95 ± 7.72	22.48 ± 9.07	−8.46 ± 5.93	<0.0001***	29.65 ± 9.44	21.82 ± 9.06	−7.83 ± 5.53	<0.0001***	0.699

a Values are presented as mean ± SD: analyzed by paired *t*-test.

b Values are presented as mean ± SD: analyzed by independent *t*-test.

*** p < 0.001.

#### Ointment Application

The mean amount of ointment used in the experimental group increased by 2.50 ± 7.63 g at Week 4 compared to baseline, and the frequency of use increased by 3.00 ± 12.17. Meanwhile, the amount of ointment used in the control group increased by 11.85 ± 21.93 g, and the frequency of use increased by 7.33 ± 11.63. There were no statistically significant differences between the two groups in the amount or frequency of ointment use. However, there was a trend for a less amount of ointment use in the experimental group compared to the control group (*p* = 0.08; Table 4).

**TABLE 4:** Changes in the amount and frequency of ointment use.

	Experimental group (n = 22)				Control group (n = 27)				*p*-value^b^
	Baseline	Week 4	Change value	*p*-value^a^	Baseline	Week 4	Change value	*p*-value^a^	
Frequency of use (times)	3.68 ± 3.98	6.18 ± 8.70	2.50 ± 7.63	0.139	5.93 ± 8.81	13.26 ± 14.55	7.33 ± 11.63	0.003**	0.100
Amount of use (g)	7.59 ± 10.83	10.59 ± 13.00	3.00 ± 12.17	0.260	6.59 ± 14.35	18.44 ± 21.68	11.85 ± 21.93	0.009**	0.081

a Values are presented as mean ± SD: analyzed by paired *t*-test.

b Values are presented as mean ± SD: analyzed by independent *t*-test.

** p < 0.01.

#### Body Measurement

The mean height of the children in the experimental group was 146.43 ± 16.20 cm at baseline, and the mean growth after medication was 0.57 ± 0.65 cm. The mean height of the children in the control group was 146.80 ± 11.93 cm at baseline, and the mean growth after medication was 0.07 ± 0.26 cm. In statistical analysis, the height of the experimental group and the control group at baseline did not show a statistically significant difference. However, at Week 4, the experimental group showed a significant level of height growth, while the control group did not show any significant change. As a result, the experimental group showed significantly more growth than the control group (*p* < 0.05). On the other hand, there were no significant differences between the two groups in changes in body weight or BMI (Table 5).

**TABLE 5:** Changes in children’s height and weight.

	Experimental group (n = 14)				Control group (n = 15)				*p*-value^b^
	Baseline	Week 4	Change value	*p*-value^a^	Baseline	Week 4	Change value	*p*-value^a^	
Height (cm)	146.43 ± 16.20	147.00 ± 16.10	0.57 ± 0.65	0.006**	146.80 ± 11.93	146.87 ± 11.78	0.07 ± 0.26	0.334	0.015*
Weight (kg)	40.21 ± 10.52	40.53 ± 10.73	0.31 ± 0.95	0.239	45.91 ± 14.30	46.47 ± 14.38	0.56 ± 1.66	0.212	0.632
BMI (kg/m^2^)	18.56 ± 2.82	18.55 ± 2.89	−0.01 ± 0.39	0.946	20.84 ± 4.86	21.09 ± 4.83	0.25 ± 0.74	0.205	0.243

a Values are presented as mean ± SD: analyzed by paired *t*-test.

b Values are presented as mean ± SD: analyzed by independent *t*-test.

* p < 0.05.

** p < 0.01.

In the analysis of the children and their parents, such as the parents’ age, parents’ education level, parents’ occupation, family income, weight and height at birth, breastfeeding experience, which can affect height growth, no statistically significant difference was observed between the experimental group and the control group (Table 6). In addition, residential life (type of resident, residential environment, construction period, new/repaired house or new furniture, etc.), eating habits and other matters (cooking method, whether to take dietary supplements, number and type of eating out, type of snack, the number of exercise, participants’ ethnicity etc.) did not show any significant difference between both groups (Table 6).

**TABLE 6:** Analysis of influencing factors related to children’s height growth.

			Experimental group (n = 15)	Control group (n = 15)	*p*-value
General characteristics of parents	Parent’s age	Under 30	1 (6.67%)	0 (0.00%)	1.000^a^
		30–34	1 (6.67%)	1 (6.67%)	
		35–39	4 (26.67%)	4 (26.67%)	
		Over 40	9 (60.00%)	10 (66.67%)	
	Education level	High school graduate	5 (33.33%)	7 (46.67%)	0.456^b^
		College graduate	10 (66.67%)	8 (53.33%)	
	Occupation	Employed	11 (73.33%)	12 (80.00%)	1.000^a^
		Unemployed	4 (26.67%)	3 (20.00%)	—
	Family income (US dollor)	Under 200	2 (13.33%)	1 (6.67%)	0.582^a^
		200–300	1 (6.67%)	2 (13.33%)	
		300–400	6 (40.00%)	3 (20.00%)	
		Over 400	6 (40.00%)	9 (60.00%)	
General characteristics of children	Weight at birth (kg)	—	3.34 ± 0.38	3.26 ± 0.49	0.592^c^
	Height at birth (cm)	—	50.37 ± 1.60	49.93 ± 1.98	0.519^c^
	Breastfeeding experience	No lactation	3 (20.00%)	2 (13.33%)	0.775^a^
		Colostrum lactation	2 (13.33%)	2 (13.33%)	
		Under 6 months	6 (40.00%)	4 (26.67%)	
		Over 1 year	4 (26.67%)	7 (46.67%)	

a Values are presented as mean ± SD or number (%): analyzed by Fisher’s exact test.

^b^Values are presented as mean ± SD or number (%): analyzed by chi-square test.

c Values are presented as mean ± SD or number (%): analyzed by independent *t*-test.

### Safety Evaluation

#### Adverse Reaction

The adverse reactions reported by the participants were mild abdominal pain (two persons), acute palmoplantar vesicular eczema (one person), and fever and coughing (one person). Of these, the eczema and fever and coughing were considered to be unrelated to the prescribed drug, and disappeared promptly after monitoring. The two cases of abdominal pain were both in the placebo group, and one of these patients showed recovery without separate treatment. The other patient withdrew their consent and dropped out of the clinical trial due to the abdominal pain.

#### Diagnostic Test and Vital Sign Monitoring

In blood tests and vital sign monitoring after 4 weeks of medication, pulse and TSH levels of the children in the experimental group showed a significantly greater decrease from baseline compared to the control group (*p* < 0.05). The body temperature, SBP, DBP of the children in the experimental group also showed a decrease compared to the baseline. However, this change in TSH and vital sign was within the normal range, and all study participants did not show any abnormal signs in laboratory tests or vital sign monitoring throughout the study (Table 7).

**TABLE 7:** Changes in children’s TSH and Vital sign.

	Experimental group (n = 14)				Control group (n = 15)				*p*-value^b^
	Baseline	Week 4	Change value	*p*-value^a^	Baseline	Week 4	Change value	*p*-value^a^	
TSH (uIU/ml)	1.46 ± 0.48	1.21 ± 0.36	−0.25 ± 0.32	0.012*	1.40 ± 0.68	1.89 ± 1.23	0.49 ± 1.08	0.103	0.022*
SBP (mmHg)	100.86 ± 10.73	100.79 ± 10.36	−0.07 ± 10.20	0.980	103.93 ± 14.56	106.27 ± 15.10	2.33 ± 11.33	0.438	0.554
DBP (mmHg)	59.93 ± 7.15	59.57 ± 9.47	−0.36 ± 12.07	0.911	63.00 ± 11.96	63.27 ± 12.04	0.27 ± 8.42	0.904	0.872
Body temperature (°C)	36.79 ± 0.21	36.78 ± 0.32	−0.01 ± 0.38	0.890	36.67 ± 0.22	36.77 ± 0.22	0.09 ± 0.28	0.224	0.396
Pulse (times/min)	85.57 ± 5.46	82.36 ± 5.88	−3.21 ± 8.34	0.146	81.33 ± 6.79	83.67 ± 5.05	2.33 ± 5.14	0.100	0.039*

a Values are presented as mean ± SD: analyzed by paired *t*-test.

b Values are presented as mean ± SD: analyzed by independent *t*-test.

* p < 0.05.

## Discussion

AD is a relapsing, pruritic, inflammatory skin disease, reported to be caused by interactions between the immune system, microbial exposure, genetic and environmental factors, and skin barrier dysfunction (Flohr and Mann, 2014; Otsuka et al., 2017). For treatment of AD, depending on the etiology and the severity of symptoms, methods such as avoidance strategies, topical anti-inflammatory therapy, and phototherapy are used (Wollenberg et al., 2018a; Wollenberg et al., 2018b). Of these, application of steroidal ointment is the most commonly recommended treatment method to alleviate symptoms (Arellano et al., 2007). However, long-term steroid use can cause adverse reactions such as acne, steroid rosacea, telangiectasia, perioral dermatitis, and atrophy. For patients who do not respond to steroid treatment, AD can recur (Fukaya, 2000; Atherton, 2003).

The incidence of AD has been gradually increasing over several centuries, and currently affects around 20% of the world population (Bantz et al., 2014). The need to develop new therapeutic agents has become prominent due to the increasing socioeconomic costs of AD (Carroll et al., 2005; Suh et al., 2007), and this has been accompanied by growing interest in the potential of alternative medicine.

SCRT is a herbal medicine that has been used in East Asia to treat diseases related to the allergic march, such as allergic rhinitis and asthma, for hundreds of years (Shin et al., 2018). SCRT is reported to have an “anti-allergic effect” (Ikeda et al., 2002) and an “anti-inflammatory effect” (Shin et al., 2018). However, to date, there have been almost no clinical studies investigating the effects of SCRT for AD.

With the aim of verifying the efficacy of SCRT for AD, this study evaluated efficacy and safety in patients taking SCRT or a placebo. In the efficacy assessment, there were no statistically significances between the two groups in objective, subjective, and total SCORAD scores. This is thought to be due to the effects of the steroidal ointment supplied to all participants to provide the minimal treatment.

There were also no statistically significant differences between the two groups in the change in amount and frequency of ointment use. However, the experimental group showed a trend for less ointment use than the control group (*p* = 0.081). The reason for this phenomenon seems to be that the previously reported anti-allergic effect such as inhibiting IL-4 and CD4^+^ of SCRT and its ingredients (Ikeda et al., 2002) affected the progression and remission of atopic dermatitis. This indicates that medication with SCRT could reduce AD patients’ dependency on steroidal ointment.

In anthropometric analysis only including children (participants aged <18 years), the experimental group showed a significantly greater increase in height from baseline to Week 4 than the control group (*p* < 0.05). Most of the children in this study belonged to the school-age (7–12 years old), and in the statistical analysis conducted on these participants (13 from the experimental group and 12 from the control group), the experimental group showed a statistically significant height growth compared to the control group (*p* = 0.016). The remaining five participants (two from the experimental group and three from the control group) belonged to adolescence (over 13 years old), and in the height growth, no statistically significant difference was shown between the experimental group and control group. However, because the number of adolescence participants was too small, it was judged to have no statistical significance. In addition, as a result of investigating and comparing factors that can affect height growth such as parents’ socioeconomic environment, physical information of the children, residential life, eating habits, participants’ ethnicity and exercise, the experimental group and the control group didn't show any statistically significant differences in these items. This suggests that SCRT has a significant effect as a growth promoter for children with AD.

In blood tests and vital sign monitoring after 4 weeks of medication, TSH levels of the children in the experimental group showed a significantly greater decrease from baseline compared to the control group (*p* < 0.05). Thyroid hormones are known to have a major effect on children’s growth and development (Tarım, 2011). The fact that the experimental group of this study showed height growth and TSH reduction after 4 weeks of SCRT administration suggests that there may be some association between SCRT, TSH, and children’s height growth. However, considering the fact that the relationship between SCRT and TSH has not been clearly identified, further studies are needed to clarify this association.

In adverse reaction monitoring, vital sign monitoring, and laboratory tests to evaluate the safety of SCRT, there were no abnormal findings. Considering that previous studies have reported allergic diseases, such as allergic asthma and AD, leading to short stature in children (Ferguson et al., 1982; Kristumundsdottir and David, 1987; Snat’Anna et al., 1996), the growth-promoting effects of SCRT are thought to be mediated not only by hormones, but also by amelioration of allergic disease. The results of this study provide evidence to support claims that SCRT is a safe treatment method without adverse effects that can be used in children with AD as an alternative to growth hormone, which is controversial.

The mechanisms behind the growth-promoting effects of SCRT are unclear, and other factors affecting growth such as parental height could not be actively controlled. In addition, it is difficult to generalize the results as the number of research participants analyzed for height growth is relatively small. However, if independent studies of SCRT or a long-term, large-scale study of the effects on AD relief and growth factors such as T3, T4, and IGF-1 were performed, the mechanisms could be revealed more precisely.

## Conclusion

The experimental group that took SCRT showed a trend for reduced dependence on steroidal ointment compared to the control group. In addition, SCRT treatment resulted in a significant promotion of height growth in children with AD. Also in safety evaluation, all study participants did not show any abnormal signs including laboratory tests or vital sign monitoring throughout the study.

## Data Availability Statement

The original contributions presented in the study are included in the article/supplementary material, further inquiries can be directed to the corresponding author.

## Ethics Statement

The studies involving human participants were reviewed and approved by The institutional review board of the Wonkwang University Korean Medicine Hospital. Written informed consent to participate in this study was provided by the participants’ legal guardian/next of kin.

## Author Contributions

JL: Conceptualization, Visualization, Writing of the original draft. EJ: Methodology, Revision and editing of the draft. JJ: Methodology, Revision and editing of the draft. Y-EK: Methodology. M-JS: Methodology. SK: Resources. GY: Resources. YS: Data curation, Resources. MP: Conceptualization, Project administration, Supervision, Revision and editing of the draft.

## Funding

This work was supported by a grant of the National Research Foundation of Korea (NRF) that was funded by the Korean government (MSIP 2008-0062484) (2015M3A9E3051054).

## Conflict of Interest

The authors declare that the research was conducted in the absence of any commercial or financial relationships that could be constructed as a potential conflict of interest.
